# Women's preferences for NIPT as a first‐line test in England and France: Challenges for genetic counseling practices

**DOI:** 10.1002/jgc4.1839

**Published:** 2023-11-16

**Authors:** Adeline Perrot, Angus Clarke, Carine Vassy, Ruth Horn

**Affiliations:** ^1^ Ethox Centre University of Oxford Oxford UK; ^2^ Institute of Medical Genetics Cardiff University Cardiff UK; ^3^ Institute of Interdisciplinary Research on Social Issues University of Sorbonne Paris Nord Bobigny France; ^4^ Ethics in Medicine University of Augsburg Augsburg Germany

**Keywords:** ethics, non‐invasive prenatal testing, policy, prenatal genetics, women's experiences

## Abstract

Non‐invasive prenatal testing (NIPT) is provided in the private and public sectors worldwide as a first‐ or second‐tier test. In England and France, NIPT is fully funded and offered as a contingent strategy with different probability cut‐offs (1:150 and 1:1000). These different approaches to define the target population for NIPT have implications for how women experience their antenatal care. The paper explores and compares the perceptions and difficulties of women in England and France who took NIPT as a second‐tier screening test. It is based on a semi‐structured qualitative interview study with 17 women in England and France conducted between September 2021 and May 2022. The interviews were cross‐analyzed using thematic analysis. Our findings show that most women express a preference for the offer of NIPT as a first‐line screening test. Some issues with the contingent model, related to the access to information and termination of pregnancy (TOP), the disparities of NIPT uptake, and risks of generating anxiety with combined first‐trimester screening (cFTS), could be addressed by a universal strategy for T21, T13, and T18. Nevertheless, this strategy could present some challenges for genetic counseling due to: women's understanding and expectations of NIPT; adequate information and counseling about the scope and limits of NIPT; concerns about the routinization of NIPT in the first‐line offer; limitations and uncertainties associated with the provision of expanded NIPT in France; the remaining importance of other screening tests; and associated costs.

**What is known about this topic?**

NIPT as a first‐tier screening test is preferred by pregnant women in several countries. NIPT is the best‐performing screening test that can be offered in the first line for T21, T18, and T13.

**What this paper adds to the topic?**

This paper sheds light on the contexts of contingent NIPT in England and France, the implications with regard to differing probability cut‐offs, and women's experiences in each country. The paper highlights the preference of women interviewed for a universal strategy and the challenges this raises for counseling practices.

## 1 INTRODUCTION

Non‐invasive prenatal testing (NIPT) is a screening test available in the private sector worldwide (Minear et al., 2015) and provided as a first‐ or second‐tier test in the public health systems of some countries. In recent years, the Netherlands and Belgium have developed a universal strategy of offering NIPT as first‐tier screening in a genome‐wide approach with a partial contribution from pregnant women (Lannoo et al., 2023; van der Meij et al., 2021). In contrast, England and France propose a contingent strategy of fully funded NIPT following combined first‐trimester screening (cFTS) from differing probability cut‐offs: 1:150 in England and 1:1000 in France for T21, T13, and T18 (and for other rarer aneuploidies in France) (Perrot & Horn, 2022). These different choices with regard to probability cut‐offs and hence the target populations who may be eligible for NIPT free of charge have a direct impact on patients’ experiences of their antenatal pathways. This paper proposes to compare the perceptions, difficulties, and preferences of women in England and France in relation to the current offer of NIPT as a second‐tier screening test. It is based on qualitative in‐depth interviews with women (*n* = 17) in both countries.

The comparative analysis between England and France highlights similarities and contrasts in the experiences of women interviewed in relation to each country's contingent program. Each model has its advantages and disadvantages, which are important to consider with regard to women's preference for earlier, universal access to NIPT. From the perspective of the women, NIPT as a first‐line screening test carries the advantage of earlier access to test results, reduced waiting times, lower levels of anxiety as well as access to earlier termination of pregnancy (TOP). We discuss the universal strategy and highlight that, while NIPT as a first‐line screening test may address some of the current issues, it nevertheless raises questions about the challenges for counseling practices.

### 1.1 NIPT as a contingent strategy in England and France

The choice of offering NIPT as a contingent test in England and France, rather than a first‐tier screening test, is mainly based on health‐economic considerations (HAS, 2017a, 2017b; Morris et al., 2014). Both health systems fund NIPT under certain eligibility conditions to a population of pregnant women who, following a positive result on cFTS, are estimated to have a “high chance” (England), or “high risk”/“increased risk” (France) of a fetal anomaly. These national programs express different priorities within each public health system.

In England, the introduction of NIPT, in June 2021, highlighted a strong focus on reducing the number of invasive procedures and associated risks of miscarriage compared to the previous NHS Down's Syndrome (DS) screening (Trisomy 21) program (Morris et al., 2014), and on maintaining a similar or lower cost (Hill et al., 2014; Morris et al., 2014). The more cost‐effective option of a 1:150 cut‐off was chosen over a 1:1000 cut‐off involving a slightly, yet not significantly higher detection rate (Hill et al., 2014). This appears to reflect the consensus position that was adopted in England following a public consultation by the UK National Screening Committee (UK NSC) to “have minimal impact on the expenditure on the screening programme compared to alternatives”(UK National Screening Committee (UK NSC), 2015). Therefore, the UK NSC chose to keep the number of invasive procedures low (UK National Screening Committee, 2015) rather than increasing the detection rate (sensitivity of the test) of fetuses with DS. In France, a different strategy was adopted. French health authorities chose to allocate additional resources (17.5 M€ per year) (HAS, 2017a, 2017b) compared to the 2015 screening strategy (cFTS) to improve the “performance” of prenatal screening: increasing the detection rate for DS and reducing the number of false negatives and positives (HAS, 2017a, 2017b).

### 1.2 Methods

This paper is part of a comparative research exploring the ethical practical issues arising from the introduction of NIPT into clinical routine care in England, France, and Germany. The broader qualitative study includes a literature review and semi‐structured interviews with health professionals, women, and other stakeholders (policy‐makers, associative stakeholders) involved in the use, discussions, and/or decisions about NIPT.

This present paper is based on interviews conducted with women in England and France to capture their perceptions and experiences of NIPT.

#### 1.2.1 Recruitment

In France, women were recruited via a well‐known health and well‐being forum (Doctissimo) and the sharing of the invitation to participate in the study via midwives in private practices (providing NIPT, either reimbursed publicly or performed privately “for personal convenience”). In England, women were recruited by posting the invitation on the websites of patients’ organizations providing information about NIPT, such as the Down Syndrome Association, Antenatal Results and Choices and Unique (Understanding Rare Chromosomes and Gene Disorders). Invitations were posted only once on each website and then remained on the websites.

Interested women and/or couples then contacted us via email to arrange a date for an interview. After each interview, we asked the interviewee to further share our invitation email with other persons they knew had made experience with NIPT. Due to our strategy, we do not know how many women/couples received our invitation and responded to it. We pursued this recruitment strategy over a year until we felt was had a reasonable insight into the experiences women/couples in each country made with NIPT.

The inclusion criteria were any women and/or couples who had been confronted with the decision to undergo or not NIPT. In France, we recruited 8 women, all of whom were between 30 and 40 years old, had French surnames, and lived in urban areas. All participants were well educated and occupied a position of responsibility, with 5 out of 8 requiring higher education qualification. In England, we recruited nine women, four of whom were between 30 and 40 years old and five between 40 and 50 years old. All participants had English surnames, lived in urban areas, and had higher education qualifications. Seven women occupied highly qualified positions, one woman was a part‐time charity worker and parent carer, and one woman a “stay‐at‐home‐mum”.

The recruitment was undertaken by AP and RH (PI), two experienced qualitative researchers trained in the social sciences.

#### 1.2.2 Data collection

Prior to the interviews, interviewees were provided with a participant information sheet detailing the purpose of the study, the funding body, and the institutional affiliation and role of the researchers. On the day of the interview, consent was obtained to conduct, record, and transcribe the interviews; use anonymized quotes in scientific publications; store de‐identified transcripts, and deposit these in the UK Data Archive. Consent was obtained online by reading the consent form out aloud and asking the interviewee whether they agree or not. A copy of the consent form signed by the interviewer was then emailed to the interviewee for their records.

The interviews were conducted online via Microsoft Teams in English (by AP and RH) or in French (by AP). In all but one case, the interviews were done with women, one at a time; only in one case, the woman asked her partner to join the interview. The interviewers did not know the participants prior to the interviews. The interviews were of approximately 45 minutes duration each. The interviews were digitally recorded and transcribed verbatim. Interviews were conducted between September 2021 and May 2022 with women who took the test in 2020 and 2021.

The interview topic guide (see supplementary file) was developed based on previous literature‐based research on the ethical issues of NIPT, and more specifically regarding its clinical implementation. Topics covered during the interviews ranged from experiences with: the clinical consultation, the information received and the discussion around NIPT, the circumstances of the offer, the decision whether to carry out the test, adequate time for reflection, the consent process, communication and discussion around the results, the advantages and disadvantages of the program, the medical processes and the ethical issues around NIPT. The semi‐structured interviews allowed women to describe their experiences and the different stages and events they went through, and to focus the discussion on what was important for them. While all topics from the topic guide were covered during the interviews, the order of these topics and the questions asked were adapted to each interviewee.

Once data was collected, the participant's name was replaced by a unique participant number (pseudonymization via a linkage list). The password‐protected list of participants’ names and contact details is accessible only to AP and RH (PI) and will be kept for at least 3 years after publication or public dissemination and then will be destroyed.

Ethics approvals have been obtained from the University of Oxford Central Research Ethics Committee (R64800/RE001) in the United Kingdom, and the Inserm Ethics Evaluation Committee (Inserm Ethics Evaluation Committee (CEEI)/Institutional Review Board (IRB): Avis n°21–82), France.

Data are available from the UK Data Archive (DOI 10.5255/UKDA‐SN‐856508) for researchers who meet the criteria for access to confidential data.

#### 1.2.3 Data analysis

Following a thematic analysis approach (Braun & Clarke, 2006), the interviews were coded first separately and then cross‐coded by AP and RH. The collaborative coding involved regular meetings between the researchers to discuss and review the construction of the meaning of the codes, in general, and with regard to their cultural and linguistic translatability, and their applicability to each country's data set. By doing so, a master codebook was developed and applied to the interview data using NVivo software.

We wrote memos to develop the analysis and to generate and develop themes through constant comparison with the data. The process of comparison involved the identification not just of similarities and differences in the content of what was discussed, but also examining more deeply how interviewees articulated and framed their experiences and perspectives. This required the researchers to build on their own positionality (particularly in terms of cultural, social, and linguistic background) to more meaningfully interpret the data within the context it was drawn from, and thus identify key points of comparison between the French and English data (Manohar et al., 2019). Data were then de‐identified to protect the privacy of interviewees while retaining context and content as much as possible.

It is important to note, for understanding the limitations of the study, that the vast majority of participants interviewed (16/17) reported a positive experience of NIPT. Most of these women who had a positive experience received NIPT free of cost through the public health systems (13/16); only 3 paid privately upon a “high‐chance” cFTS result (2/16) before NIPT was reimbursed or to get an earlier test at 10 weeks (1/16). This overall positive experience may be due to the fact that they did not experience false positives or negatives with NIPT that would have been revealed later by a diagnostic procedure. In almost all cases (except one), the testing was for trisomy 21 (or DS), which does not account for the types of experiences women would have reported in the event of identifying T13/T18 or rarer aneuploidies.

## 2 RESULTS

### 2.1 Motivations to undertake NIPT

Women in our interviews perceived the uptake of NIPT as a choice. Many of them said that they had done it without hesitation and that, if they had to do it again, they would do it in the same way – whether they planned to continue or terminate their pregnancy if a fetal abnormality was detected.

“[We had] no hesitation in doing the test. (…) There were no questions about it from me, from my partner either (…) I think it was our personal conviction too, my partner and I to know.” (Woman, 30–35, France, Chemical scientist)

“I think the decision was straightforward for us to carry on with NIPT actually. Considering the high score that we got with the combined test, alongside the raised NT [Nuchal Translucency].” (Woman, 40‐45, England, Speech and language therapist)

Many of the women interviewed were willing to pay for NIPT, particularly in England where the test was not publicly funded until June 2021 (or only funded as part of the implementation study RAPID) for all women with a “high chance” result. In order to access the test and have earlier “reassurance” about a “safe” pregnancy, participants paid between £250 and £450. For some of them, this meant having to borrow money from their families.

“(…) then after that [cFTS] they did [at the hospital], then say: ‘there is this other test, a blood test called NIPT but you’d have to pay for it’, and it was a lot of money. (…) £400. But, in that moment, in that time, it seemed like the better option, despite the cost. We have family that we knew could loan us the money or give us the money, so we decided to go for the NIPT because I could face that procedure because it was non‐invasive and it didn’t carry any risks to the child.” (Woman, England, 40–45, Charity worker)

“I was offered it, in this pregnancy, because I was at ‘intermediate risk’, I could have refused it if I wanted. So, I know that if I have a future pregnancy and I’m at low risk, I think I’ll do it. If it is not reimbursed, I will pay for it.” (Woman, France, 30–35, Chemical scientist)

Many women expressed their willingness to pay for the test, even if it was not available publicly or if they were in a “low chance” category.

### 2.2 Delays and waiting times

Women interviewed generally expressed positive opinions about this “simple” screening test, especially when the NIPT turnaround is short (3–10 days). The earlier time of testing compared to cFTS and the rapid turnaround for results was seen as crucial in relation to a possible decision to terminate the pregnancy. This was particularly the case in France where there is a 14‐week limit to access “voluntary” termination of pregnancy (*interruption volontaire de grossesse, IVG*) (“Article L2212‐1 du Code de la Santé Publique,” 2022; “LOI n° 2022‐295 du 2 mars 2022 visant à renforcer le droit à l’avortement (1),” 2022). After 14 weeks, the termination of pregnancy for medical reasons (*interruption médicale de grossesse, IMG*) can be done without a time limit, but only if approved by a multidisciplinary team in a specialist center for prenatal diagnosis.

“(…), it's very hard to say what I’m going to say, it's very easy to say when you’re not in this situation, but I’d rather have an abortion before three months than an IMG at six months of pregnancy. From the moment your belly starts to come out, you can feel it [the fetus] moving.” (Woman, France, 30–35, Press officer)

Women in both countries found it difficult to cope with the sequence of lengthy waits between the various screening tests. Almost all of them felt that the provision of NIPT, as a second‐line procedure, increased the waiting time before they received a definitive result from a diagnostic procedure (amniocentesis or CVS). Several women raised issues related to the lack of “accuracy” of cFTS to test for the common trisomies when comparing against NIPT. They did not understand why cFTS is still offered when a “better option,” NIPT, in their view, is available. Some women would have preferred to have NIPT as a first‐line test (around 10 weeks) to avoid this waiting period between each test and to obtain a more accurate result than cFTS more quickly.

Three women, who had already experienced either a TOP or the birth of a child with DS, insisted on the importance of avoiding seeing the fetus on the screen during the first scan (carried out at around 11–13/14 weeks in England and France).

“Afterwards, it's up to each individual, but I would find it much better, because in the end, with the ‘tri‐test’ [cFTS], it's a step. I would have preferred to have NIPT directly, it would have reduced the waiting time, in fact. Because there's this wait for the ‘tri‐test’ and the wait for NIPT, it's 3–4 weeks in total, a whole month of pregnancy waiting, and I find that very hard.” (Woman, France, 30–35, Press officer)

“Then, fast‐forward to December, I found out I was pregnant again and, this time, it was a very different experience being pregnant after having experienced loss previously (…), cause it's just fraught with anxiety every single minute, and I decided I couldn’t wait for the combined screening. I just went ahead with an NIPT at 10 weeks – I paid for that privately and this time, thankfully, we’ve come back low risk for all three and it also gave me reassurance (…).” (Woman, England, 40–45, Marketing manager)

### 2.3 Outcome of NIPT

In France, all women interviewed were “falsely” alerted following the cFTS. They were categorized as “high risk” (risk greater than or equal to 1/50) or “increased risk” (risk between 1/1000 and 1/51) and then received a screen‐negative NIPT result. In England, a very different group was interviewed, with 5/9 women having received “positive” results from NIPT. This test enabled them to decide whether to terminate the pregnancy following CVS or amniocentesis (2/9) or to prepare for the birth of an affected child (3/9) (two of the interviewees in England voluntarily paid for the test privately to avoid the invasive procedure).

Also, in England, two women paid for a NIPT test for their second pregnancy, after their first child was born with DS despite them having been in a “low chance” category. They wondered why a “low chance” cFTS result did not give them access to a free NIPT test, as even though “low”, there was still some possibility of a fetal anomaly.

“And, I was like, well if it's much [more] accurate why are you bothering with the nuchal fold test anymore? Why would you bother doing something that's 75% accurate when you have something that's 99% accurate? That was my point, I was like: ‘why are you offering?’ Like, I could wash my clothes with a washboard in a bucket, or I could wash them in a washing machine, you know, technology has moved on. I just don’t understand why the technology [NIPT] just doesn’t become part of their standard offering.” (Woman, England, 35–40, Statistician)

Also, other interviewees in both countries associated the test with a high “99% reliability”, although they acknowledged that NIPT is not a diagnostic test.

### 2.4 Preference for NIPT

In general, women interviewed had high expectations of NIPT and expressed a strong preference for this test over cFTS or more invasive procedures (CVS or amniocentesis). The interviews showed that the choice to undergo NIPT is driven either by the desire to prepare for the arrival of a child with a genetic condition earlier and without the associated risks of other, so‐called “invasive” tests (e.g., miscarriage, infection, Rhesus sensitization) (NHS, Page last reviewed: 12 October 2022; NHS, Page last reviewed: 03 January 2023), or to prepare for a decision to terminate the pregnancy. Women interviewed found it “reassuring” that the higher accuracy of NIPT compared to cFTS reduces the likelihood to undergo further invasive procedures and to obtain an earlier result. This was particularly the case for women who were concerned about pregnancy complications and did not want to take any risk for the fetus.

“I thought it was good, in fact, to be able to at least really remove concerns without having to undergo an invasive examination which, for me, can be very anxiety‐provoking and which also involves risks.” (Woman, France, 35–40, Civil servant in national cultural heritage administration)

“Between the Panorama and Harmony [NIPT] we were fairly satisfied, and all the extra ultrasounds, we were satisfied that for us, for our peace of mind, we didn’t want to go further with any sort of invasive testing. We just opted to know insofar as we could, to cover as many bases even though it's only screening.” (Woman, England, 35–40, Statistician)

### 2.5 Critical voices regarding cFTS

In France, due to the low cut‐off of 1:1000, more women than in England are offered NIPT following cFTS. Interviewees reported stress and anxiety generated by the “false” alarm and preferred a more reliable and straightforward test than cFTS at the outset. This particularly concerned women who had a history of fetal anomaly in a previous pregnancy. The anxiety experienced was related to the waiting period, which is lengthened by the different screening tests. In England and France, some women spoke of the feeling of “wasted time”, frustration and unnecessary stress or worry caused by cFTS when there are no confirmed fetal anomalies and the child turns out to be healthy.

“I really wonder how much it's worth stressing mothers, parents in general, for a risk that, when you rationalise it, is still very low. I had about a 1:500, so it was certainly a bit higher than the general population, but it wasn’t 1:10, so the risk was still very low. I feel like I was robbed of two weeks of pregnancy when I was thinking: ‘if it turns out my baby has a problem’.” (Woman, 30‐35, France, Marketing manager)

“It was as quick as it could be, really but, knowing what I know now, NIPT is amazing. I think the combined screening is good, but it can cause so much unnecessary worry for people that haven’t got a problem! And so, in an ideal world, I think… at least particular groups – people like me who’ve been through something like that before, or who are already at a higher risk because of age, or history, or whatever – it would be a wonderful thing to have that on the NHS anyway, (…) to have that choice and have that reassurance through NIPT.” (Woman, 40–45, England, Marketing Manager)

Interviewees emphasized that technology has evolved and questioned why cFTS is still offered if NIPT provides better and more straightforward results.

## 3 DISCUSSION

The main result of our study is that women interviewed would prefer an offer of NIPT as a first‐tier screening test. This is consistent with the results of other studies (Birko et al., 2019; Lannoo et al., 2023; Lewis et al., 2016; van der Meij et al., 2023). The discussion will examine the current implications of different policies and the difficulties experienced by women with regard to a contingent offer of NIPT in each country; their preferences for a first‐tier screening; and the challenges a universal strategy raises for genetic counseling.

### 3.1 Implications of different cut‐offs providing access to NIPT free of charge

In England, NIPT is funded in the case of “high chance” results at a cut‐off of 1:150. This implies a lower detection rate of conditions in fetuses (in particular T21) (Chitty et al., 2016) than in France where the cut‐off is at 1:1000 and more women have second‐tier screening. Also in our interviews, two English women, and a friend of one English participant, experienced how the restricted provision of NIPT means that some aneuploidies are not detected before birth. The UK NIPT implementation study (RAPID) estimated that the first‐line strategy would detect 289 more fetuses with trisomy for fewer procedure‐related miscarriages in comparison to cFTS, but at a higher cost than the current two‐stage strategy (Morris et al., 2014; UK National Screening Committee, 2015). Due to the restricted reimbursement of NIPT, some women who are not eligible for the test through the NHS, turn to the private sector when they want greater reassurance. This defeats the aim of introducing NIPT into the national fetal anomaly screening program which was, among others, to reduce inequalities (Nuffield Council on Bioethics, 2017). The current offer of NIPT free of charge to women with a “high chance” result only may therefore increase disparities between women who know about the test and have the financial resources to pay for it, and those who do not have these possibilities.

In France, the probability cut‐off of 1:1000 results in around 11% of pregnancies being labeled as at “high” or “increased risk” (HAS, 2017a, 2017b). This threshold was chosen because, according to estimates of the Haute Autorité de Santé (HAS), it allows to increase the number of detected trisomies (by about an additional 79–120 cases for T21). The HAS has emphasized an egalitarian approach when insisting “on the need to guarantee pregnant women equitable access to appropriate information and quality support.” (HAS, 2017a, 2017b). However, due to the low test specificity of cFTS, adopting a low probability cut‐off to access NIPT, means that many more women will undergo the test and receive more false‐positive results, which also generates more anxiety (Vassy, 2011, 2022). According to the 2017 HAS report (HAS, 2017a, 2017b), the prevalence of DS is 27.3 for 10,000 pregnancies and the positive predictive value (PPV) of the cFTS is only 5.6%. NIPT as the first‐line test will, therefore, identify many fewer women to be invited back to consider further testing than cFTS.

### 3.2 Women's preference for a one‐step screening strategy

In comparison to a two‐step approach (cFTS and NIPT), the first‐line strategy seems to have some clinical advantages such as fewer invasive tests and procedure‐related miscarriages, and a higher detection rate of trisomies (Kostenko et al., 2019). The universal strategy—which was desired by women in our interviews—may have the advantage of providing substantially higher predictive values than cFTS at an earlier stage and reduce prenatal anxiety and its negative impacts on the woman's and fetuses’ health (Allison et al., 2011). According to our interviews and other studies, earlier results would allow for precious time to either bond with the fetus (Allison et al., 2011; Katz Rothman, 1988) or prepare for terminating the pregnancy. Women interviewed emphasized the importance of earlier results especially in France where they preferred to have access to “voluntary” termination of “pregnancy” before 14 weeks (“Article L2212‐1 du Code de la Santé Publique,” 2022; “LOI n° 2022‐295 du 2 mars 2022 visant à renforcer le droit à l’avortement (1),” 2022), instead of “medical” termination of pregnancy requiring a referral from at least four professionals (Ameli. Assurance maladie, 2023) in a specialist center. Several authors (Ameli. Assurance maladie, 2023; Davies et al., 2005) have stressed the importance of the possibility of early termination of pregnancy to limit the psychological consequences, the risks of depression and harm in women, as well as for health safety and costs (Rose et al., 2022). This concern could be addressed by offering NIPT as a first‐tier screening test rather than waiting until the second trimester of pregnancy when many women start talking about it with their family and friends and feel the movements of the fetus.

### 3.3 Challenges of first‐tier screening strategy for genetic counseling

#### 3.3.1 Managing women's expectations

As we have seen, women have high expectations toward the test often associated with a “99% accuracy” for the three common trisomies. As few pregnancies are affected by an autosomal trisomy, for example, T21, one would be 99% “accurate” if one gave a low chance result to all pregnant women, without even performing a test. In order for women to make informed decisions about their pregnancy, it will be important to communicate the proportion of positive test results that are true positives (PPV); these are given as 91.78% for T21, 65.77% for T18 and 37.23% for T13 in first‐tier NIPT, although these will vary somewhat in different populations with age distribution and details of the screening program (International Society for Prenatal Diagnosis, 2018)).

#### 3.3.2 Providing information and counseling support following NIPT results

The study highlights a difference of opinion between pregnant women who wish to avoid invasive procedures that they consider as “too risky” (Dungan et al., 2023; Hill et al., 2012), and their health professionals who advocate the importance of diagnosis and, therefore, the use of an invasive procedure (Rose et al., 2020). With regard to common trisomies, the invasive procedure is perceived by health professionals as still being important (e.g., in case of false positives generated by NIPT and for the possibility of finding other fetal anomalies when performing karyotyping) (Benachi & Vivanti, 2022).

In France, diagnostic testing is not mandatory but the Association of French‐speaking cytogeneticists (2015) strongly recommends it as an “imperative” step before a TOP (Association des cytogénéticiens de langue française, 2020). The High Authority for Health (2017) also stresses that NIPT “does not replace fetal karyotyping for the diagnostic confirmation of fetal trisomy 21” (HAS, 2017a, 2017b). Also, the access to TOP for women in France from 14 weeks can be performed after the confirmation by two physicians of a multidisciplinary team and the opinion of the advisory committee that: “the continuation of the pregnancy poses a serious threat to the woman's health, or there is a strong likelihood that the unborn child will suffer from a particularly serious condition recognised as incurable at the time of diagnosis.” (“Articles L2213‐1 à L2213‐5 du code de la santé publique”).

Similarly, in England, according to the UK National Screening Committee recommendation (2016), the invasive test is “required” (Committee, 2016). In English law, however, a woman can request TOP before 24 weeks, without fetal indication, on the ground that “the continuance of the pregnancy would involve risk (…) of injury to the physical or mental health of the pregnant woman”.

Recent evidence from a variety of countries shows that since NIPT has been implemented, fewer women undergo diagnostic tests (Dungan et al., 2023; Rose et al., 2022). This could mean several things: first, since NIPT performs better than previous screening tests, fewer women receive a positive test result indicating the need to undergo further invasive procedures. Second, women might prefer to rely solely on the NIPT result and avoid a cascade of further testing, either because they do not want to put the pregnancy at risk, or they may want to choose an early TOP.

In order to allow women to make informed decisions about these options, it is important to provide good counseling about the scope and limits of NIPT. This indeed requires continuous education of professionals who provide the test, and of whom not all are specialists in genetics. Adequate support and counseling are needed also for those women who prefer to take up further diagnostic tests and who will have to wait and endure the uncertainty for further weeks (CVS and amniocentesis are carried out from 11 and 15 weeks respectively). This waiting time is complex and experienced as stressful for women, requiring good information and appropriate genetic counseling support to mitigate anxiety.

In addition, adequate communication about the limitations of NIPT is particularly important where genome‐wide NIPT is used to detect abnormalities beyond the three common trisomies (e.g., in France) (Perrot & Horn, 2022). At this point in time, however, there is a lack of evidence of test performance of expanded NIPT for other aneuploidies such as rare autosomal trisomies (RATs) and Copy Number Variants (CNVs) (Dungan et al., 2023; Hui et al., 2023).

#### 3.3.3 Routinization of screening by a universal offer

There are concerns that offering NIPT to all women as a first‐line option might increase the risk of routinizing NIPT (Ghiasi et al., 2023), hence compromise their reproductive autonomy (Birko et al., 2018). However, studies from the Netherlands, where NIPT is available as a first‐tier screening and where there is also a strong focus on women's right not to know as well as to know, show that Dutch women feel entitled to refuse the test (the uptake rate was about 46% in 2019) (Lannoo et al., 2023; van der Meij et al., 2023). This highlights the importance of a non‐directive or “reproductive deliberation” approach (Warton et al., 2023) that leaves options open and provides genetic counseling that supports the decision‐making capacity, values, and autonomous choices of women.

#### 3.3.4 Communicating the limitations of expanded NIPT in France

Several women in France stressed that they would have liked to have had access to extended results, that is, screening for other aneuploidies beyond the three common trisomies, as expanded NIPT (called also genome‐wide NIPT (GW‐NIPT)) is now available (Agence de la biomédecine, 2021; Perrot & Horn, 2022) and reimbursed at the same price as standard NIPT (360€) in the public sector of this country (Réseau Périnatalité Eure et Seine‐Maritime, 2019). However, it is not offered by all prescribers (midwives, gynecologists, GPs). Access routes are not well known and depend on the individual choices of health professionals to offer this test, which is much discussed and debated in France and within the international medical and scientific community (Perrot & Horn, 2022).

If expanded NIPT is offered as a first‐line test to detect other aneuploidies, test performance will be less satisfactory with lower or uncertain values for test sensitivity (the proportion of the autosomal trisomy cases that are detected) and its PPV (the proportion of screen‐positive cases that are true positives). The approach of counseling for health professionals should also be manageable in the context of increasing data complexity and limited counseling resources.

More and more women are likely to be alerted that a chromosomal abnormality may be present in the fetus, which may have implications in their experience of the pregnancy (e.g., difficulties in bonding with the fetus, stress while waiting for a definitive diagnosis). In the case of otherwise “uneventful” (Ville, 2019) or “low risk” pregnancies, this may change women's perception of their pregnancy and generate “unnecessary” anxiety, as discussed by Rothman in her book, “The Tentative Pregnancy” (Katz Rothman, 1988). It will be important to weigh the risks and benefits of offering expanded NIPT to pregnant women to avoid harm in reporting findings for other abnormalities. Women should receive good quality pre‐test counseling so that they can understand the possible implications of GW‐NIPT, be prepared to the feedback of potentially rarer abnormalities than the three common trisomies, and determine whether or not they want these extended data to be generated.

#### 3.3.5 Considering the complementary role of other screening tests

Furthermore, we would like to emphasize that there is no evidence that NIPT as a first‐tier test should replace other screening tests such as the cFTS or ultrasound, as they can indicate fetal anomalies (e.g., neural tube defects, skin defects) and pregnancies at risk of preeclampsia and intrauterine growth restriction (Hill et al., 2014; McLennan et al., 2016; Nicolaides et al., 2013). This will involve clear communication about the place of NIPT in a broad screening pathway.

#### 3.3.6 Costs considerations

Finally, although the majority of women interviewed would prefer NIPT as a first‐line screening test, studies point out that the universal strategy is not yet cost‐effective (García‐Pérez et al., 2018; Jayashankar et al., 2023; Ravitsky et al., 2021), and this regardless of the level of risk in women (García‐Pérez et al., 2018). According to current cost–benefit analyses, a contingent provision strategy remains the most cost‐neutral so far (Ravitsky et al., 2021). However, few analysis consider the potentially cost‐saving impact of a positive result on post‐natal management such as better and quicker access to surgical specialists for neonates with heart defects.

## 4 CONCLUSION

The women interviewed in England and France were critical of the offer of NIPT as a contingent strategy in their country and asked for a first‐line access. They reported their need for reassurance, the stress caused by high rates of false‐positive and false‐negative results of the cFTS, the long delay before having a definitive diagnosis of a genetic anomaly, and the unnecessary anxiety during their pregnancies.

This raises the question of whether a universal strategy would be a better option, able to address the limits of the contingent test strategy. It seems that a first‐line provision of NIPT to detect the most common trisomies (T21, T13, and T18) might be appropriate (International Society for Prenatal Diagnosis, 2018), whereas for rarer aneuploidies, at least at this point in time, the universal offer might increase anxiety and the number of inconclusive or inaccurate results. In addition, this strategy could present several challenges in terms of resources available for genetic counseling. It would be important to provide balanced, clear, unbiased, and transparent communication about the meaning, scope, and limitations of NIPT. This should help to prevent the risks of routinisation of the offer, increased anxiety in pregnant women, and misunderstanding about the possibilities of this screening test.

However, there is some evidence that NIPT as a first‐tier test allows for earlier reassurance and/or earlier diagnosis enabling women to either prepare for a child with a genetic condition or earlier access to TOP. When considering women's perspectives, providing as much “peace of mind” (Bowman‐Smart et al., 2019) as possible seems important to support their reproductive choices, as long as women receive high‐quality counseling.

### 4.1 Limitation of our study

The majority of women we interviewed had a positive experience of NIPT, lived in urban areas, were well educated, and occupied highly qualified positions. For a more nuanced analysis, it would be important to interview more women and recruit women from more diverse social, economic, and ethnic backgrounds and levels of literacy. Further research is also needed to explore the attitudes of women who report a negative experience with this screening test (e.g., due to false negative/positive results) and use these results to encourage further discussion on the use of NIPT, improve clinical and counseling practices, and relevant guidelines. Also, it would be crucial to investigate the experiences of women who received results for rarer aneuploidies in order to understand how perceptions of the test are likely to differ between targeted and genome‐wide NIPT.

## AUTHOR CONTRIBUTIONS

The study was designed and funding was obtained by RH. Data were collected and analyzed by RH and AP who conceptualized this manuscript. Initial drafting and coordination of further drafts was performed by AP. All authors (AP, AC, CV, RH) were involved in feedback and further drafting. All authors were involved in reviewing, editing, and approving the final version of the manuscript for submission.

## FUNDING INFORMATION

This research is funded by the UK Economic and Social Research Council (ES/T00908X/1).

## CONFLICT OF INTEREST STATEMENT

The authors declare no conflict of interest.

## ETHICS STATEMENT

Human studies and informed consent: Ethics approvals have been obtained from the University of Oxford Central Research Ethics Committee (R64800/RE001) in the United Kingdom, and the Inserm Ethics Evaluation Committee (Inserm Ethics Evaluation Committee (CEEI)/Institutional Review Board (IRB): Avis n°21–82), France. All procedures followed were in accordance with the ethical standards of the responsible committee on human experimentation 7 and with the Helsinki Declaration of 1975, as revised in 2000. Informed consent was obtained from all patients for being included in the study.

Animals studies: No non‐human animal studies were performed by the authors for this paper.

## Supporting information


Data S1.

## Data Availability

Data is available from the UK Data Archive for researchers who meet the criteria for access to confidential data: Horn, Ruth (2023). Non‐invasive Prenatal Testing Study: Comparison England, France, Germany, 2021–2022. [Data Collection]. Colchester, Essex: UK Data Service. 10.5255/UKDA‐SN‐856508. https://reshare.ukdataservice.ac.uk/856508/
